# Rapid recovery of complete mitogenome of Indian major carp, *Catla catla* from low depth paired end Illumina sequencing

**DOI:** 10.1080/23802359.2017.1298413

**Published:** 2017-03-10

**Authors:** L. Sahoo, A. Bit, P. K. Meher, K. Murmu, J. K. Sundaray, P. Das

**Affiliations:** Fish Genetics and Biotechnology Division, ICAR-CIFA, Kausalyaganga, Bhubaneswar, India

**Keywords:** Next generation sequencing, *Catla*, phylogenetics, mitochondrial

## Abstract

Here we report the reconstruction of the catla (*Catla catla*) complete mitochondrial genome sequence from low depth paired end Illumina sequencing. The genome is of 16,597 bp in size. Similar to other vertebrate mtgenomes, it consists of 13 protein-coding genes, 22 tRNAs, 2 rRNAs and a putative control region. The present mtgenome is 3 bp longer than the earlier reported catla mtgenome from our laboratory. Majority of the mitochondrial genes are encoded by the H-strand. Phylogenetics analysis revealed that *Catla catla* is closer to *Labeo rohita* than other labeo species. Present study demonstrated the power of next generation sequencing towards hassle free and rapid sequencing of mitochondrial genomes of non-model organisms.

*Catla catla* popularly known as catla is the second most important Indian major carp widely cultured in Indian subcontinent and wild stocks of catla were declining day by day (Reddy 2005). Conservation and protection of genetic diversity is essential for sustainable production of aquaculture species. Mitochondrial DNA has been successfully utilized for several population genetic studies due to its peculiar characteristics (Avise et al. 1987). Complete mtDNA sequence has several advantages over partial mtDNA sequences (Miya et al. 2003). The complete mtDNA of catla has been sequenced and reported using Sanger’s sequencing (Accession no NC_016892 and JQ_087872). However, the advantages of the next generation sequencing (NGS) technology were not explored by any of the previous study. So, in the present study, we have reconstructed the complete mitogenome of catla (Accession no KY419138) from Illumina short paired end reads and compared with complete mitogenomes of other cyprinids.

Genomic DNA from fin tissue of a single specimen (Voucher No: 1506, stored at ICAR-CIFA) of catla, collected from river Mahanadi was extracted using standard phenol-chloroform method (Sambrook & Russel 2001). Illumina paired end libraries were prepared and sequenced on an Illumina Nextseq500 (San Diego, CA) platform. Approximately 7 GB of sequence data was obtained. *De novo* assembly was performed using CLC Genomics Workbench software (CLC Bio, Aarhus, Denmark) with maximum stringency (Fraction (LF) = 0.50, Sequence Similarity (SIM) = 0.80) and minimum contig length 200 bp. In total 697,565 contigs with N50 of 643 bp were obtained. Contings ranging from 10 to 16 kb in length were subject to blast in NCBI database. The contig of 16,597 bp in size was observed to be the mitogenome of catla and annotated using MitoAnnotator (Iwasaki et al. 2013). Complete mitogenome sequences for other cyprinids were downloaded from the NCBI database. Phylogenetics analysis along with the reconstructed catla mitogenome was performed using MEGA6 (Tamura et al. 2013).

The complete mtgenome of catla was found to be 16,597 bp and comprised of 13 protein-coding genes, 22 tRNAs, 2 rRNAs and a putative control region. The present mitogenome of catla reconstructed from NGS data is largest in size in comparison to the mtDNAs reported earlier. Organization of genes in the catla mitogenome is in conformity with other vertebrates. Except few genes, majority of the mitochondrial genes were encoded in the H-strand. In all the protein-coding genes ATG was the start codon except COI which encoded GTG. Similarly, TAA and an incomplete stop codon T– – were encoded by protein-coding genes except ND3 which encoded TAG in catla. Out of 22 tRNA genes, 21 catla tRNA genes can fold into a typical cloverleaf structure, except for tRNA^Ser^ (AGY) lacking dihydrouridine arm. Length of tRNA genes ranged from 67 to 76 bp. The D-loop is 929 bp in size and contains a microsatellite (TA)_7_, a putative termination-associated sequence and three conserved sequence blocks. There were 2 overlaps and 10 intergenic spacer regions with a 36 bp long intergenic spacer region in between tRNA^Asn^ and tRNA^Cys^. Interestingly the mtgenome yielded from the NGS shared 99% similarity to the mtgenome sequenced earlier in our laboratory (Bej et al. 2012). The phylogenetic (Figure 1) analysis showed that catla is closer to *Labeo rohita.* The present study demonstrated the power of next generation sequencing, the need for PCR-free mitogenome sequencing without involving sample processing, primer designing and long range PCR in non-model aquatic species.

**Figure 1.**
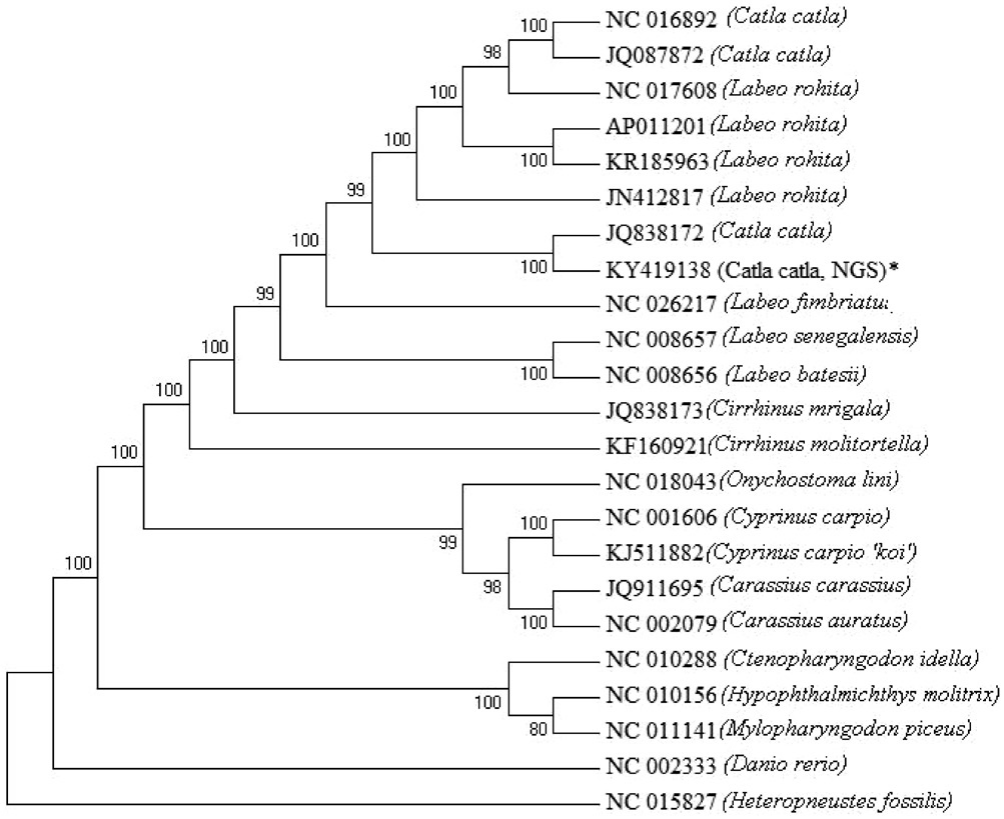
ML tree of complete mtgenome sequences of 22 teleosts.
